# Care for the chronically ill in Germany – The challenges during the COVID-19 pandemic

**DOI:** 10.25646/7168

**Published:** 2020-11-11

**Authors:** Christa Scheidt-Nave, Benjamin Barnes, Ann-Kristin Beyer, Markus A. Busch, Ulfert Hapke, Christin Heidemann, Maren Imhoff, Rebekka Mumm, Rebecca Paprott, Henriette Steppuhn, Petra von Berenberg-Gossler, Klaus Kraywinkel

**Affiliations:** Robert Koch Institute, Berlin Department of Epidemiology and Health Monitoring

**Keywords:** CHRONIC DISEASE, CARE, CARE UPTAKE, HEALTH CONSEQUENCES, COVID-19 PANDEMIC, GERMANY

## Abstract

The COVID-19 pandemic is posing major challenges to the health care sector. This scoping review compiles evidence concerning changes to health care service availability and utilisation as well as possible impacts on health for selected groups of chronically ill people in Germany. The focus is on cancer, cardiovascular diseases, diabetes mellitus and mental disorders. Most empirical data available concerned inpatient care and showed a clear decline in the utilisation of inpatient treatments in March and April 2020 in the areas of oncology and cardiology as well as in mental health. For cardiovascular emergencies such as heart attack and stroke, a decline was observed especially regarding less serious cases. Although there were indications of treatment delays, there was no evidence thus far that emergency care had been generally compromised due to adjustments to inpatient care capacities. In the outpatient setting, extensive adjustments to health care services availability were observed for all disease groups considered. Overall, very limited empirical data were available. In particular, hardly any data were available on how changes in care impacted population health. There is an urgent need for continuous surveillance and evaluation based on health care and epidemiological data.

## 1. Introduction

To contain the spread of SARS-CoV-2 infections and prevent overburdening the health care system, Germany, among other countries, implemented a set of non-pharmaceutical interventions (NPI) beginning in mid-March 2020. These included, on the one hand, measures to reduce physical contact and recommendations for social distancing [1]. On the other hand, adjustments to all areas of medical care were introduced. For example, inpatient treatment capacity, especially intensive care capacity for COVID-19 patients, was expanded and elective (postponable) surgery at all hospitals was postponed until further notice [2].

Results of international studies suggest that treatment figures for people with common noncommunicable diseases have decreased during the COVID-19 pandemic in many countries, at least temporarily. The reasons for this are manifold. Restrictions on the availability of care seem to have played as large a role as has a reluctance of patients to utilize services [3–8]. A recent World Health Organization (WHO) survey of health ministries in 160 countries [9] showed that the extent and duration of health care shortfalls are determined to a large extent by type of disease, extent of regional SARS-CoV-2 infections and by pre-existing differences in the capacities of individual countries’ health care systems to provide and adapt their services.

This article reviews changes in the health care situation of patients with selected noncommunicable diseases in Germany between the beginning of March and mid-June 2020. The focus is on cancer, cardiovascular diseases, diabetes mellitus and mental disorders. All these diseases require continuous and quality-assured care, some within the framework of structured disease management programmes (DMPs). In addition, study data show that patients with diabetes, cardiovascular diseases and some cancers may be at increased risk of developing severe symptoms in the course of a COVID-19 infection [10–13]. The following questions should be answered: (1) How have availability and utilisation of health care services changed for these groups of chronically ill people in Germany following the introduction of NPIs to control the spread of SARS-CoV-2? (2) Is there evidence that such measures have affected the health and well-being of chronically ill people? On the basis of this review, we will identify areas where there are obvious gaps in the available evidence and in which new questions arise that require continued and more in-depth work.

## 2. Methodology

This review is based on the objectives and methodological approach of a scoping review as developed by Arksey and O’Malley [14]. This form of systematic review serves to gain an overview of the state of knowledge in complex subject areas. Once the research question has been formulated, a systematic literature search is carried out and a tabular presentation and description of the identified study types, central concepts and results is prepared.

### 2.1 Research sources

To identify relevant literature, a search was conducted for peer reviewed publications in the PubMed and COVID-19_MAP literature databases. To include commentaries from stakeholders, grey literature (non-publisher-bound publications) and opinions of experts [15, 16], an additional keyword search in Google and Google Scholar and on the websites of selected organisations was carried out, and sources cited in reference lists were taken into account.

### 2.2 Search strategy

Our search covered the period from 1 March to 19 June 2020 (for Google Scholar from 2020 on) with a focus on publications pertaining to cancer, cardiovascular diseases, diabetes mellitus and mental disorders. For each of the four subject areas considered, the electronic PubMed literature database was systematically searched with four different search queries in English (the search strings used are available on request from the corresponding author). In order to ensure the greatest possible congruence in the topic-specific search, all search queries contained a core of combined search terms for SARS-CoV-2 and COVID-19. This core was linked with further combinations of search terms specific to the respective disease groups. Simpler combinations of search terms were used for the additional searches in Google and Google Scholar and for searches on the websites of relevant professional societies, professional associations and patient organisations. The literature searches were stratified by disease group and carried out by at least one person per group. At least two co-authors were involved in screening and selecting the literature search results for each disease group. Unclear or conflicting screening decisions were discussed, and consensus was reached among the co-authors involved.

### 2.3 Inclusion and exclusion criteria

All German- and English-language contributions relating to the health care of patients with cancer, cardiovascular diseases, diabetes mellitus or mental disorders in Germany were included. Contributions that related exclusively to cancer screening examinations or to persons in outpatient home nursing care or long-term residential care were excluded.

### 2.4 Data preparation and presentation of results

The included sources were stratified by disease area, and essential information was extracted and tabulated using a predefined matrix. In addition to bibliographic information, responsible stakeholders or institutions, key contents and results as well as the type of publication were documented. Regarding the type of publication, a distinction was made between empirical studies with their own data basis and non-empirical studies (e.g. commentaries, recommendations, information and communications). All results of the literature search were summarised in two tables (Table 1 and Table 2), corresponding to the two questions posed above (changes to health care services availability and utilisation, effects on health and well-being).

## 3. Results

This review covers a total of 62 publications with highly uneven numbers and types of publications for each question and disease area (Table 1 and Table 2). A total of 40 publications were identified exclusively via the additional internet-based search. The remaining 22 sources (including all sources on cancer, 7 of 24 sources on cardiovascular diseases, 2 of 18 on diabetes, and 2 of 9 on mental disorders) were found via PubMed. An additional report by the Scientific Institute of the AOK (WldO), published shortly after the end of the search period, was nonetheless included, since preliminary results of this analysis had been published in advance during the period covered by the search [17, 18].

### 3.1 Changes to health care availability

In all disease groups considered, major adjustments to health care availability have taken place (Table 1). A distinction must be made between, on the one hand, changes to the regulatory framework, such as billing modalities, and, on the other hand, recommendations by medical societies.

In the area of oncology, guidelines prioritising the urgency of surgical cancer treatments were implemented (Table 1) in order to ensure that necessary operations could be carried out promptly even when intensive care resources became limited [19, 20]. To segregate patient flows in the health care setting and reduce the risk of infection for cancer patients, it was recommended that treatments be carried out in specialised oncological facilities where possible [20, 21]. Further recommendations involved modifying treatment plans under consideration of the individual situation. For example, the use of less toxic chemotherapeutic agents was suggested and, if possible, avoiding infusions in favour of orally administered drugs [20]. In the field of radiotherapy, distributing the required radiation dose over fewer appointments was discussed as an option to reduce the number of contacts and to achieve a shorter treatment duration [22, 23].

For patients with cardiovascular diseases, the pandemicrelated adjustments also led to restructuring of inpatient care capacities. For example, hospital beds in dedicated wards for patients with acute stroke (stroke units) or with acute chest pain (chest pain units) were partly converted into intensive care beds [24, 25]. According to the medical societies, however, the care of patients with cardiovascular diseases could still be fully assured [26–28]. In addition, by simplifying approval procedures for rehabilitative follow-up treatment, transfers to rehabilitation clinics were accelerated [26, 29]. However, medical professional associations have reported that invasive examinations and procedures (e.g. cardiac catheterisation and stent implantation) were sometimes only carried out after a two- to three-week waiting period and that an increasing number of patients had to visit more distant clinics [30]. Moreover, cardiologists in private practices increasingly took over treatment of patients who otherwise would have been admitted as inpatients for observation [30]. According to the German Society of Cardiology (DGK), the call to postpone elective operations resulted in a significantly lower number of heart operations carried out overall, including the number of more urgent elective operations that should normally have been carried out within 30 days [31].

Routine diabetes care was also curtailed to provide capacity for COVID-19 patients [32, 33], including the postponement of elective surgical procedures (e.g. kidney transplantation and bariatric surgery) [34–36]. In addition, the changes made by the Federal Joint Committee (G-BA) to the DMP requirements guideline were particularly important for the treatment of patients with cardiac insufficiency or diabetes. To avoid SARS-CoV-2 infections, patient training sessions, which are normally compulsory parts of the DMP and are usually carried out in a group setting, as well as the documentation of medical examinations by a doctor, were suspended during the first to third quarters of 2020 [37]. In order to ensure that people with diabetes continued to receive training, video training was considered a viable option [32, 38–41] with some health insurances reimbursing such services [42, 43]. Individual training and consultations remained possible, subject to compliance with hygiene regulations [38].

Inpatient admissions to psychiatric (day) clinics or rehabilitation facilities were restricted and appointments for treatment postponed. In order to protect mental health care availability for people experiencing acute crises, inpatient services were replaced by outpatient services. Outreach services and treatments were made available to people in need of a high level of support to cope in their daily lives, to those who have difficulty obtaining necessary medication and to those who lack sufficient social support [44].

Medical societies and professional associations recommended expanding telemedicine services for patients and establishing video consultation hours [20, 45–48]. Regarding mental health, for example, these recommendations impacted both regular psychotherapeutic treatment and crisis intervention. For the treatment of physical diseases, follow-up care and check-up appointments as well as tumour conferences have been conducted using telemedicine approaches. These changes were also made possible by expanding billing options to include telemedicine and video consultations [49]. Certain certified video service providers could also be used free of charge in April/May [45]. In psychiatry, new services such as telepsychiatric crisis services, special crisis hotlines or other local services were set up [50].

### 3.2 Changes in care provision and utilisation

The number of hospital admissions of cancer patients insured by the statutory AOK health insurance decreased significantly in the period from mid-March to the beginning of April 2020 (calendar weeks 12 to 14) compared to the same period in the previous year (Table 1) [17]. The figures for oncological operations were mixed: for some cancer diagnoses (especially colorectal and lung cancers) the number of primary operations fell by around 20%, while for other diagnoses either no significant decreases or even increases (breast and cervical cancers) were recorded. The number of second operations (breast and colorectal cancers) fell by more than 70% [18]. A survey of nuclear medicine departments and practices, mainly from Germany, concerning cancer and cardiovascular diagnostic and therapeutic procedures also showed a decline in tumour diagnostic procedures of between 14% and 58% (depending on examination type). In contrast, radiation therapy figures for malignant tumours have remained stable [51]. For cardiovascular diseases, the same study showed an overall decrease in myocardial scintigraphy in outpatients, with a more pronounced trend for hospitals compared to radiological practices [51].

Analyses of AOK insurance data showed a significant decrease in hospital admissions for acute ischemic stroke, myocardial infarction or heart failure in March/April 2020 compared to the corresponding period in the previous year [17]. In a more in-depth analysis, this decrease was particularly marked for mild cases of heart attack and stroke [18]. The same applied to the admission of patients with an abdominal aortic aneurysm [18]. In concordance with these results, decreases in inpatient treatments for heart attack and in acute treatments for heart failure and arrhythmias were reported for the early phase of the pandemic based on data from a second statutory health insurance company (the Deutsche Angestellten Krankenkasse, DAK) and two hospital networks [52–54]. One university hospital in southern Germany recorded significantly fewer admissions due to minor acute heart attacks – those without changes in the ECG typical of an infarction (NSTEMI) – in March/April 2020 than during the same period in 2017, 2018 and 2019 [55]. The number of admissions due to more severe acute myocardial infarction with ECG changes typical of an infarction (STEMI) did not change, however. Consistently fewer emergency treatments than in previous years were also observed for patients with minor stroke or transient ischaemic attack (TIA). This is shown by analyses of data from 36 regional stroke centres in the Ruhr area [26] and from several stroke units at university hospitals [56], as well as from the Alfried Krupp Hospital in Essen [26]. In addition, a decline in neurovascular emergencies was also observed at the Charité Berlin during the early phase of the pandemic [57].

Furthermore, the associations of statutory health insurance physicians and professional associations have reported a reduction in the number of consultations in oncology and cardiology practices, with similar developments being reported in the areas of endocrinology and diabetology [32, 33, 58–62]. Many professional societies as well as patient organisations urged patients not to neglect the treatment of their chronic disease for fear of infection and, for example, to schedule and attend follow-up and treatment appointments [27, 28, 36, 63]. There were also warnings against independently discontinuing medication such as ACE inhibitors, which are used to treat high blood pressure and were for a while suspected of increasing the risk of a severe course of COVID-19 [64].

The WldO institute’s analysis of AOK insurance data showed a 49% decrease in inpatient treatment for mental and behavioural disorders during the stricter phases of the lockdown compared to the previous year [65]. The Central Institute of Mental Health in Mannheim recorded a 27% decrease in the use of its emergency service for people with mental health issues, particularly for affective disorders [66]. However, the German Association for Psychiatry, Psychotherapy and Psychosomatics (DGPPN) reported that the 450 psychiatric outpatient clinics in Germany observed an increase in the number of patients from residential and semiresidential care facilities [67].

### 3.3 Changes to health care services and their impacts on health

So far, very little empirical data exist concerning the impacts on health due to the observed changes to health care service availability and utilisation. Instead there are predominantly opinions on the feared consequences of delayed diagnosis and therapy, as well as results from surveys of patients and doctors (Table 2).

Cancer patients often expressed concerns about potentially inadequate or delayed treatment. The possibility of becoming infected and the risk of suffering a severe course ofCOVID-19 due to cancer or therapy-related immunosuppression have also caused uncertainty [20, 68]. Access restrictions for visitors and accompanying persons of patients in inpatient and outpatient care were considered a burden [68, 69]. In oncological clinical research, there were concerns about delays in recruitment of patients to ongoing clinical therapy trials [20]. These play an important role in providing care to people with rare cancers in particular. A task force formed by the German Cancer Aid, the German Cancer Society and the German Cancer Research Centre (DKFZ) warned of a ‘wave’ of oncological treatments as a result of the lockdown and changes made necessary by the pandemic [70].

According to Germany’s Association of Cardiologists in Private Practice (BNK), some cardiology patients who cancelled appointments were later treated as emergencies [61]. Furthermore, the German Cardiac Society has also reported an increase in the incidence of complications typical of untreated heart attacks [31]. Based on data from the University Hospital of Ulm, the blood values of heart attack patients, which can provide information about the extent of the organ damage caused, during the early stages of the pandemic were compared with those from the corresponding periods in the years 2017 to 2019 [55]. It was found that in the current year higher average concentrations of high-sensitivity troponin T (hsTnT) were measured than in previous years, which may indicate a delayed onset of therapy [55]. The Charité hospital in Berlin also found indications of delayed treatment for patients with chronic subdural haematoma (collection of blood between the outer covering of the brain (dura mater) and the brain) [57]. For example, patients admitted during the early stages of the pandemic showed more severe symptoms and a worse prognosis in the hospital setting [57].

Various uncertainties were observed among people with diabetes. Patients with diabetes were worried that they might face a higher risk of developing and suffering a severer course of COVID-19 and that they might be infected with SARS-CoV-2 during a doctor’s appointment [60]. Patients were afraid of shortages in diabetes medication [71] and reported problems with the contact-free transmission of therapy data to their diabetology practice and uncertainty about how to adjust insulin requirements under changing everyday conditions [72]. The risk of stigmatising people with diabetes as a COVID-19 risk group by excluding them from public life was also discussed [73].

## 4. Discussion

Our research on the changes to the health care situation for people with selected noncommunicable diseases in Germany during the first months of the COVID-19 pandemic identified only a few empirical studies that were almost exclusively related to inpatient care. The analyses of AOK-insurance data showed a clear decline in inpatient admissions for oncology in March/April 2020 [18]. Other European countries have reported similar declines in hospital admissions and registered cancer diagnoses since mid-February [8, 20, 74, 75]. For cardiovascular diseases, in line with international reports [5, 6, 74, 76–78], both AOK data and data from cardiological and neurological clinical departments indicate a decline in the number of patients receiving emergency medical or acute diagnostic care in the same period compared to the previous year [79]. This was associated primarily with a decrease in the number of mild heart attacks and strokes as well as transient ischaemic attacks. According to an analysis of data from 36 emergency departments in Germany, a sharper decrease was likewise observed in less urgent compared to more urgent emergency treatments following implementation of lockdown measures [79].

In conjunction with some evidence from international studies, this pattern can be interpreted as indicating a substantial role of patients reducing or delaying utilisation of health care services [18, 26, 55–57]. An expert report on the effects of the COVID-19 Hospital Relief Act also supports such a hypothesis. In their final report, the members of the committee conclude that a patient’s decision to go ahead with treatment or not has played a greater role in explaining the decline in inpatient treatment figures in 2020 compared to the same period last year than hospitals cancelling treatment [80]. For example, in the period from January to May 2020, the proportion of emergency hospital admissions was higher for the first time than the proportion of non-emergency admissions, and the number of less urgent treatments decreased more than more urgent ones [80]. However, our review only identified two empirical studies for Germany indicating a delayed utilisation of medical care for heart attack and chronic subdural haematoma [55, 57]. Moreover, only a few of the articles identified in the search period discussed outpatient care. According to reports from the National Association of Statutory Health Insurance Physicians (KBV), significantly fewer appointments in oncological and cardiological practices were made during the early stages of the pandemic [61]. An analysis by the Central Research Institute of Ambulatory Health Care in Germany (Zi) has confirmed these observations, but was published outside the review period. According to this analysis, treatment figures in oncological, cardiological, neurological, endocrinological, psychiatric and psychotherapeutic practices fell in the course of March 2020 by up to 40% in the last week of March compared to the same period in the previous year [81].

Our research identified no results from either quantitative or qualitative studies on the reasons for a delayed utilisation of inpatient or emergency medical treatment in Germany. We were also unable to identify any population-based quantitative or qualitative studies that report whether and why outpatient appointments did not take place (i.e. whether they were cancelled or postponed, either by the doctor’s practice due to lack of capacity or by the patients themselves). This question will require further research. Some reports identified by our review indicated that (partial) closures of and limited admissions to hospitals had to be compensated by outpatient services, for example in outpatient cardiology [30] and psychotherapy [82]. Results from ongoing research projects monitoring outpatient care since March 2020, such as COVI-Prim (Accompanying Monitoring of Primary Care in General Practitioners’ Practices during the COVID-19 Pandemic), would be important for gaining a deeper insight into the complex requirements that outpatient care providers had to meet, especially in the early stages of the pandemic [81, 83].

Ongoing analyses of the developments of health care availability and utilisation in Germany for specific groups of chronically ill persons are important, especially if a new spike in cases were to develop in the course of the pandemic. For example, professional societies in the field of oncology began, at an early stage, to develop a wide range of recommendations to adapt procedures in order to avoid interruptions to diagnostic and therapeutic measures that cannot be postponed while minimising the risk of infection. One potential problem was that a backlog of patients could still develop, partly because older patients in particular may have avoided the health care system, despite symptoms, and thus not have received prompt diagnostics and treatment [20, 70]. Temporary suspension (mammography screening) or reduced utilisation of cancer screening examinations could also contribute to this.

Encouraged by recommendations from medical societies and associations as well as new billing options, tele-medical care services for patients with chronic physical illnesses and mental disorders were expanded. There is great potential for further development of these options beyond the end of the current pandemic [20, 39, 40, 84]. Further analysis is required to determine the extent to which these services are accepted and used by medical professionals and patients. Such analyses should identify barriers to utilisation due to deficiencies in technical equipment and expertise as well as the inherent limits of such services [72, 85, 86].

According to the results of surveys, expert assessments and reports from the field, the COVID-19 pandemic has not led to critical bottlenecks in oncological care in Germany, and the system has overall been perceived as being relatively adaptable. Probably only a small number of time-critical therapies were postponed. In contrast to European regions with high COVID-19 case numbers, no fundamental restructuring of emergency care for stroke patients in Germany has so far appeared to have occurred [26]. According to the medical societies, the timely diagnosis and treatment of patients with acute cardiovascular events has at no time been at risk [27, 28]. Despite the closure of diabetes units as part of the adaptations of inpatient care capacities in the context of the pandemic [32, 33, 38], it has been assumed for the time being that the care of diabetes patients could be largely maintained, at least in paediatrics [38]. The health care situation for people with mental disorders cannot yet be fully assessed.

The key question as to the extent to which patients with chronic diseases have suffered damage to their health as a result of pandemic-related changes to health care availability and reduction in utilisation cannot be answered at present. Our review identified only two empirical studies that found suggestive evidence of delayed utilisation followed by greater disease severity in patients with heart attacks and elderly people with chronic subdural haematoma [55, 57].

We cannot rule out that delays in diagnostic assessments and follow-up appointments, also due to changes in the utilisation patterns of patients, have led to a shift of diagnosis to later disease stages. Such a shift could lead to poorer treatment outcomes with increases in acute complications or longer-term sequelae [31, 48, 55, 57, 61, 70, 87, 88]. For example, an article published after the literature review period showed that the frequency of diabetic ketoacidoses in children and adolescents with newly diagnosed type 1 diabetes was higher during the early stages of the pandemic than in the comparable period of previous years, which could be due to a delay in diagnosis [87]. As a result of the COVID-19 pandemic, experts expect an increase in mental disorders such as adjustment disorders, anxiety disorders, depression and trauma disorders [89] to which the health care system will have to respond in the longer term. Based on past experiences, the German Association for Psychiatry, Psychotherapy and Psychosomatics also points out that increased suicide rates must be anticipated, especially if the economic downturn caused by the pandemic continues to worsen [90].

In summary, these results show a considerable need for research both on the causes and the consequences of changes to the availability and utilisation of health care services by chronically ill people in Germany.

### 4.1 Strengths and limitations

One of the strengths of the present review is that it included not only a structured search in PubMed but also additional searches via the search engines Google and Google Scholar as well as the websites of selected organisations. The expanded search enabled us to identify the vast majority of contributions, including important reports from the WldO institute on changes in hospital case numbers in March/April 2020 as well as opinions, recommendations for action and information from medical societies and professional organisations. In addition, the searches were specifically targeted at four common noncommunicable disease groups for which changes to health care availability and utilisation related to the pandemic are particularly relevant. The most important limitation is that gaps remain in the review; for example, unpublished results, ongoing work or work in publication were not systematically researched by contacting the relevant institutions. Similarly, as a scoping review, only a categorisation according to type and content of cited sources was conducted, with no evaluation regarding scientific validity. Our searches only covered the period between March and June 2020, meaning that results published outside the search period could not be included in the systematic overview, even if they refer to the period between the beginning of March and mid-June 2020.

### 4.2 Conclusions

In summary, during the early stages of the COVID-19 pandemic the number of people treated for cancer, cardiovascular diseases, diabetes mellitus and mental disorders decreased in Germany. In orderto learn from this for future crises, the roles played by changes to health care availability and declines in utilisation will require further clarification. In order to make necessary adjustments quickly, it will be important to continue monitoring the health care situation throughout the ongoing course of the pandemic. Only a targeted monitoring of the developments in outpatient and inpatient care can shed light on adverse and possibly long-term consequences for patient’s health and well-being due to changes in the health care situation. At the population level, the close monitoring of the development of cause-specific morbidity and mortality will require a reliable, timely and continuously available data basis.

## Key statements

- The number of inpatient treatments for people with cancer, cardiovascular diseases and mental disorders fell sharply in March/April 2020 in Germany compared to the same period in the previous year.
- There were no indications that emergency care for stroke and heart attack had been comprised by adjustments to inpatient care capacity in Germany.
- A decrease in hospital admissions for stroke, heart attack and other cardiovascular emergencies has been presumed to be associated with delayed utilisation of available services.
- Decreasing outpatient treatments for cancer, cardiovascular disease and diabetes mellitus could have been due to changes in availability and utilisation of health care services.
- The health care situation of people with chronic physical and mental illnesses must continue to be analysed on an ongoing basis, particularly with regard to implications for health.

## Figures and Tables

**Table 1:** Publications on changes to the health care of chronically ill persons in Germany (search period 1 March 2020 to 19 June 2020) (a) Changes to the availability of health care Source: Own table

Type of publication* and source	Participating institutions	Contents/results
**Cancer**		
1 [69]	International Society of Geriatric Oncology (partner institution in Germany: Heidelberg University Hospital)	Changes to the provision of care for older people with cancer in ten countries including Germany. Findings included an increase in telemedicine services, postponement of surgical procedures, infection monitoring, restricted access to care facilities for accompanying persons and visitors
1 [91]	Nuclear medicine facilities, international (participating institutions in Germany: Albert Ludwig University Freiburg, Ludwig Maximilian University Munich, University Hospital Essen)	Recommendations and experiences with changes to care in radiation medicine facilities, including work organisation (separate care teams), infection monitoring (symptomatic screening), prioritisation of patients with progressive disease, suspension of elective studies and treatments
1 [92]	German Society for Haematology and Medical Oncology (DGHO)	Highlights the special situation of cancer patients, recommendations on, among other things, ensuring adequate care, infection monitoring, separation of patient flows
1 [20]	Cancer Core Europe (CCE) consortium (participating institutions in Germany: German Consortium for Translational Cancer Research (DKTK), German Cancer Research Centre (DKFZ), National Center for Tumor Diseases (NCT))	Experiences at participating institutions in the face of the pandemic and recommendations for adjusting care in the centres of the Cancer Core Europe network of hospitals, including work organisation, disease management, patient counselling, and research
2 [51]	Nuclear medicine institutions in Germany, Austria and Switzerland (participating institutions in Germany: University Hospital Essen, Centre for Radiology and Nuclear Medicine Rhineland)	Survey among institutions on changes to health care conditions and availability/procedures in nuclear medicine, including work organisation, diagnostics, therapy
2 [23]	European Breast Cancer Research Association of Surgical Trialists (EUBREAST) (participating institutions in Germany: Charité – Universitätsmedizin Berlin; Brustzentrum Esslingen (BZE))	Survey of breast cancer centres worldwide, including more than 30 facilities in Germany, on the impact on care and adjustments to disease management, including PCR screening before admission, shortening of radiation and systemic therapy, postponement of surgery; significant extension of the time between diagnosis and start of treatment
1 [19]	Thoracic Surgery Outcomes Research Network (ThORN) (participating institution in Germany: University of Cologne)	Recommendations for prioritising interventions
1 [22]	Ludwig Maximilian University Munich (LMU), Technical University (TU) Munich, Institute for Radiation Medicine (IRM) Neuherberg, German Consortium for Translational Cancer Research (DKTK), University Hospital Freiburg, University Hospital Zurich, Medical University Innsbruck	Recommendations for radiooncology, including work organisation, infection monitoring and hygiene
1 [48]	University Medical Center Hamburg-Eppendorf (UKE)	Experiences with changes to the conditions under which care is provided and adjustments to care since the start of the pandemic at the UKE, including in the areas of outpatient care, inpatient care, clinical research and prioritisation of treatment
1 [21]	Martini-Klinik at the University Medical Center Hamburg-Eppendorf (UKE)	Experiences of a prostate cancer centre regarding changes to care since the start of the pandemic, including occupancy of intensive care beds, screening of patients before surgery
**Cardiovascular diseases**		
1 [47]	Professional Association of German Neurologists (BDN)	Recommendation to reduce physical patient contacts, adaptation of the organisation in the reception area, establishment of telephone and video appointments, relief for medical treatment chains and clinics
1 [46]	German Cardiac Society (DGK)	Recommendation to limit direct patient contact by making use of telemedicine or to restrict contact to those patients who need to be seen by a doctor
1 [45]	Federal Association of Cardiologists in Private Practice (BNK)	Information on possibilities for offering telemedical care, use of certain certified video service providers free of charge in April/May 2020
1 [24, 28]	German Society for Neurology (DGN)	Call to maintain evidence-based care for patients with cerebrovascular diseases including guideline-based secondary prevention and neurological rehabilitation, report on partial temporary conversion of stroke unit beds into intensive care beds, stroke care in German hospitals nonetheless continues to be provided without restrictions
2 [25]	University of Duisburg-Essen, University Hospital Heidelberg, University Medicine of Johannes Gutenberg University Mainz, 261 certified Chest Pain Units (CPUs) in Germany	Survey in CPUs showing that 97% of CPUs were affected by federal and state level pandemic plans regarding structural redistribution and modification of bed capacity; university clinics increased (+4%), teaching hospitals (-3%) and other providers (-9%) reduced the number of CPU beds
1 [27]	German Cardiac Society (DGK)	Plea not to ignore life-threatening heart disease- availability of emergency care is assured
1 [31]	German Cardiac Society (DGK)	Assessment that emergency care for patients with acute heart disease has been maintained
1 [26]	Alfried Krupp Hospital Essen, Heinrich Heine University Düsseldorf Ruhr University Bochum	Assessment that no fundamental restructuring of emergency medical and acute diagnostic stroke care (including certified stroke units) has taken place, few stroke unit closures due to local outbreaks, rehabilitative care has been maintained
1 [93]	German Society of Neurology (DGN)	Information on changes to procedures for follow-up rehabilitation to a procedure of direct admission by hospitals
1 [30]	Federal Association of Cardiologists in Private Practice (BNK)	Report that invasive examinations and procedures (e.g. cardiac catheters, stent implantations) were sometimes only carried out after a waiting period of two to three weeks or were then scheduled at extremely short notice (on the same day), lack of capacity led to patients being treated at more distant clinics, cardiologists in private practice increasingly took over the outpatient care of patients who would normally have been monitored as inpatients
**Diabetes mellitus**		
1 [37, 49]	Federal Joint Committee (G-BA)	Recommendations to suspend compulsory patient training and medical examinations in the context of disease management programmes (temporary special arrangement)
1 [38]	Practice for paediatric and adolescent medicine, diabetological practice (expert contribution)	Description of cutbacks to routine care to a necessary minimum in order to increase capacity for COVID-19 patients; Group training for children with diabetes and their families in direct contact replaced by virtual interactive training sessions; individual training and individual consultations in direct contact still possible, subject to compliance with hygiene regulations; introduction or expansion of telemedicine services with video consultation for children with diabetes and their families; relaxed restrictions on a certain number of billable sessions per patient or for certain patient groups
1 [39]	Diabetological practice (expert contribution)	Discussion of perspectives on and chances for introducing or expanding telemedicine services for adults with diabetes
1 [40]	Two university departments for paediatric diabetology, University of Hannover and University of Lisbon (expert contribution)	Discussion of perspectives on and chances for introducing or expanding telemedicine services for children with diabetes and their families
1 [32]	German Diabetes Society (DDC)	Description of cutbacks to routine care to a necessary minimum in order to expand capacities for COVID-19 patients, as well as the discontinuation of in-person group training for patients with diabetes; explanation of possibilities and necessary conditions for telemedicine services
1 [33]	German Diabetes Society (DDG)	Communication from the professional society on the effects on inpatient care structures in diabetology, shifting of available capacities up to the closure of diabetes departments in hospitals to provide care to COVID-19 patients
1 [42]	Practice for general medicine (expert contribution)	Information on the extension of billing options for telemedicine, certain billing codes can be reimbursed in addition to video appointments
1 [43]	Practice for general medicine (expert contribution)	Information on extending billing options also for patients with private health insurance through special regulations in the medical fee schedule (GOÄ)
1 [34]	International consortium of experts	Information on postponing elective procedures, e.g. bariatric surgery
1 [35]	German Society for Internal Medicine (DCIM)	Information on postponing elective procedures, e.g. kidney transplantations
1 [94]	National Association of Statutory Health Insurance Funds (CKV)	Information on options to provide patients with medical devices, preferably by mail order, and transmitting prescriptions from medical practices directly to mail order businesses
**Mental disorders**		
1 [44]	Centre for Psychiatric Rehabilitation, University Psychiatric Services Bern; University Hospital for Psychiatry and Psychotherapy, University of Bern; Department of Health, Bern University of Applied Sciences	Report on the impact on mental health care structures, temporary (partial) closures of wards and services, limited access to (day) clinics and limited availability of therapeutic and other contact persons
1 [82]	German Association for Psychiatry, Psychotherapy and Psychosomatics (DGPPN)	Communication by the medical society on the postponement of treatment in psychiatric hospitals and the replacement of inpatient services with outpatient services to ensure care in acute crisis situations
1 [95]	National contact and information point for the motivation and support of self-help groups (NAKOS)	Information on changes to available care services in support of self-help groups for mentally ill persons, self-help contact points were closed and only accessible by telephone/email, group meetings or personal exchanges were not possible
1 [96]	Federal Chamber of Psychotherapists (BPtK)	Information on possible switches to telephone and video appointments as well as online interventions for treatment and therapy
1 [50]	Federal Chamber of Psychotherapists (BPtK)	Information on the expansion of existing telephone services and the development of new services and more specific local therapy and counselling services
1 [97]	German Society for Psychology (DCPs)	Opinion from this medical society on the need to allow emergency childcare in day-care centres and schools to children and adolescents with mental health problems and of mentally ill parents if needed
**Cancer**		
2 [18]	Scientific Institute of the AOK (WldO)	Analysis of insurance data regarding changes in the number of treatments in the 2020 study period (calendar weeks 12 to 14, 16 March to 5 April 2020) compared to the same period of the previous year inpatient treatment figures with the principle diagnosis ‘neoplasms’: decrease by 22% surgical interventions (seven selected diagnoses): decrease in first interventions for colorectal and lung cancer by 22% and 20% respectively, for other diagnoses (including breast cancer and prostate cancer) significantly smaller decreases (<10%) or even increases; significant decrease in second interventions (breast and colorectal cancer) by>70%.
1 [20]	Cancer Core Europe (CCE) consortium (participating institutions in Germany: German Consortium for Translational Cancer Research (DKTK), German Cancer Research Center (DKFZ), National Center for Tumor Diseases (NCT))	Discussion of the declining number of cancer patients admitted to European cancer centres within the Cancer Core Europe network, which at the beginning of April 2020 was 70% to 80% of the expected volume
2 [51]	Nuclear medicine institutions in Germany, Austria and Switzerland (participating institutions in Germany: University Hospital Essen, Centre for Radiology and Nuclear Medicine Rhineland)	Survey of institutions on the decrease in the proportion of outpatients; no decrease in the treatment of malignant neoplasms
2 [23]	European Breast Cancer Research Association of Surgical Trialists (EUBREAST) (participating institutions in Germany: Charité – Universitätsmedizin Berlin; Brustzentrum Esslingen (BZE))	Survey of breast cancer centres worldwide, including more than 30 facilities in Germany; increase in genetic diagnostics before possible neoadjuvant treatment (treatment to reduce the size of a tumour before surgery)
1 [21]	Martini-Klinik at the University Medical Center Hamburg-Eppendorf(UKE)	Discussion of experiences of the clinic regarding the cancellation of operations by patients and the clinic, temporary slight decrease in admissions
**Cardiovascular diseases**		
2 [51]	Institutes of nuclear medicine in Germany, Austria and Switzerland (participating institutes in Germany: University Hospital Essen, Centre for Radiology and Nuclear Medicine Rhineland)	Survey in hospitals and practices showed a decrease in the number of outpatient myocardial scintigraphies carried out, with greater differences in hospitals than in private practices (survey between 14 April and 20 April 2020 on changes in the previous three weeks)
1 [31]	German Cardiac Society (DGK)	Report on a nationwide decline in heart surgeries by 60%, also in cases with more urgent indications (to be performed within 30 days)
2 [17, 18]	Scientific Institute of the AOK (WldO)	Analysis of health insurance data showed a decline in inpatient treatments in calendar weeks 12 to 14 of 2020 compared to 2019 for acute heart attacks overall (-31%), heart attacks without ECG changes (NSTEMI, -33%), heart attacks with ECG changes (STEMI, -26%), chronic ischaemic heart disease (-50%), strokes (-19%), transient ischaemic attack (TIA, -39%), heart failure (-43%), aortic dissections or ruptured aortic aneurysms (-1%), non-ruptured aortic aneurysms (-53%)
2 [54]	DAK-Gesundheit statutory health insurer	Analysis of health insurance data showed a 25% decrease in hospitalisations due to heart attacks in March 2020 compared to the figures for March in 2018 and 2019
2 [52]	Hamburg Asklepios hospitals	Analysis of inpatient data from selected hospitals showed a 39% decline in hospitalisations for heart attacks between the end of March and the end of April 2020 compared to 2019
2 [55]	University Hospital Ulm	Analysis of inpatient data showed a decrease in heart attacks without ECC changes (NSTEMI) in the period 21 March to 20 April 2020 compared to the figures for the years 2017 to 2019 with an incidence rate ratio (IRR) of 0.46 (95% CI; 0.27-0.78); no changes to heart attacks with ECG changes (STEMI), unstable angina pectoris, cardiac arrhythmia and preclinical cardiac arrest
2 [53]	Helios hospitals	Analysis of inpatient data from selected hospitals showed a 22% to 28% decrease in emergency admissions for heart failure, a 13% to 27% decrease for cardiac arrhythmias, and a 15% to 27% decrease for related interventional treatments in March/April 2020 compared to 2019
2 [56]	University of Mannheim, University Hospital Erlangen, University Hospital Dresden, University of Freiburg	Analysis of inpatient data from selected hospitals showed a 43% to 85% decrease in hospitalisations due to TIA in three of four university stroke units and a 38% to 46% decrease due to stroke in two of four centres in the period calendar week 1 to 15 of 2020 compared to 2019
2 [26]	Ruhr Neurovascular Network (NVNR) (27 stroke units, 9 neuro-interventional centres)	Analysis of inpatient data from selected hospitals showed a decrease in emergency room visits due to TIA and mild stroke in the early phase of the pandemic, in some centres a decrease in stroke treatments with reperfusion therapy
2 [26]	Alfried Krupp Hospital Essen	Analysis of inpatient data showed a 45% decrease in hospital admissions due to TIA in the period from 16 March to 19 April 2020 compared to the period from 10 February to 15 March 2020, no decrease due to ischaemic stroke/intracerebral haemorrhages
2 [57]	Charité – Universitätsmedizin Berlin	Analysis of inpatient data showed a decline in inpatient acute treatments for neurovascular emergencies and chronic subdural haematoma in the period from 1 February to 15 April of 2020 compared to 2019
1 [61]	NDR enquiry to associations of statutory health insurance physicians and dentists and professional associations	Report on a 30% to 50% reduction in appointments in cardiology practices in the early phase of the pandemic
1 [64]	German Heart Foundation (DHS)	Information on potentially anxious patients who stop taking heart medication such as ACE inhibitors and Sartane
1 [27, 63]	German Cardiac Society (DGK) and German Heart Foundation (DHS)	Call for people to seek immediate medical care in the event of typical symptoms such as heart pain, shortness of breath or tightness in the chest area
1 [28]	German Society of Neurology (DGN)	Call for people to seek immediate medical care in case of typical stroke symptoms
**Diabetes mellitus**		
1 [33]	German Diabetes Society (DDG)	Communication from the medical society on declining patient numbers in practices, outpatient clinics and emergency rooms
1 [62]	Association of Statutory Health Insurance Physicians of Bavaria (KVB)	Discussion on the decline in utilisation of practice services on the basis of billing data from Bavarian practices, particularly among specialists (depending on the speciality, 25% to 70%) and with regard to early detection services among general practitioners (-80%)
1 [41]	German Diabetes Society (DDG)	Recommendations of the medical society for diabetes management including therapeutic goals for adult COVID-19 patients with diabetes as well as for closely monitored care, whenever possible by telephone or telemedicine; recommendations for the use of diabetes medication by COVID-19 patients
1 [36]	International consortium of experts	Recommendations of the expert consortium for the screening of all COVID-19 patients for undiagnosed diabetes, for diabetes management including therapeutic goals in adult COVID-19 patients with diabetes, and for the close monitoring of COVID-19 patients (with mild course) with gestational diabetes, type 1 diabetes and concomitant diseases. Depending on the severity of course of COVID-19, recommendation to intensively monitor people with diabetes. In cases of severe COVID-19 in people with diabetes, change from oral medication to insulin treatment Postponement of elective procedures, e.g. metabolic operations
**Mental disorders**		
2 [66]	University of Mannheim	Analysis of data from the Central Institute of Mental Health on the use of their emergency service for people in mental crises, 27% decrease in uptake compared to the same period last year
2 [65]	Scientific Institute of the AOK (WldO)	Analysis of insurance data on the 49% decline in inpatient admissions for mental and behavioural disorders
1 [67]	German Association for Psychiatry, Psychotherapy and Psychosomatics (DCPPN)	Communication from the medical society on the increase in the number of patients from inpatient and semi-residential care in outpatient clinics

* 1 non-empirical studies, e.g. discussion papers, recommendations, information and communications

2 empirical studies, e.g. surveys, analyses of insurance data or inpatient data

**Table 2:** Publications on the health effects of changes to the care of the chronically ill in Germany (search period 1 March 2020 to 19 June 2020) Source: Own table

Type of publication* and source	Participating institutions	Contents/results
**Cancer**		
1 [69]	International Society of Geriatric Oncology (participating institution in Germany: Heidelberg University Hospital)	Discussion of burdens caused by access restrictions for accompanying and visiting persons at health care facilities
1 [20]	Cancer Core Europe (CCE) consortium (participating institutions in Germany: German Consortium for Translational Cancer Research (DKTK), German Cancer Research Center (DKFZ), National Center for Tumor Diseases (NCT))	Discussion of causes of concern and uncertainty among cancer patients, especially with regard to infection, severe COVID-19 courses due to immunosuppression, restrictions in health care availability; there is a strong demand for counselling services (e.g. the Cancer Information Service)
2 [68]	German Cancer Society (Working Group Prevention and Integrative Oncology (PRiO))	Survey of medical staff, patients; both groups suffer from general restrictions (especially contact restrictions) and worry about restrictions to necessary therapies with negative consequences for health; doctors fear long-term mental or physical consequences for themselves, report increased time needed for consultation; patients are aware of doctors’ heavy workload
**Cardiovascular diseases**		
1 [61]	Federal Association of Cardiologists in Private Practice (BNK)	Report that cardiology patients who had recently cancelled appointments were now being registered as emergency cases
2 [55]	University of Ulm	Analysis of inpatient data showed an increase in the average concentration of high-sensitivity TnT (hs-TnT) in heart attack patients with ECG changes (STEMI) in the period 21 March to 20 April of 2020 compared to the years 2017 to 2019
1 [31]	German Cardiac Society (DCK)	Report on an increase in the number of patients with complications typically related to untreated heart attacks
2 [57]	Charité – Universitätsmedizin Berlin	Analysis of inpatient data showed that the proportion of chronic subdural haematoma patients with more severe symptoms at admission was higher in the period 1 February to 15 April of 2020 compared to 2019 and that those patients had a worse prognosis during their time in hospital
**Diabetes mellitus**		
1 [73]	German Diabetes Society (DDG)	Communication from the professional association on the risk of stigmatisation of diabetes patients through exclusion from work, in schools and in public
1 [71]	diabetes DE – German Diabetes Aid	Communication from the organisation on the concerns felt by diabetes patients wanting sick leave without having an acute illness; concern about a lack of diabetes medication
1 [60]	Institute for Work and Technology, Westphalian University of Applied Sciences Gelsenkirchen, Bocholt, Recklinghausen	Press release on the fear of people with diabetes of being infected with COVID-19 in general practices
1 [72]	diabetes DE – German Diabetes Aid	Press release on the uncertainties of people with insulin-treated diabetes, in particular in relation to the use of continuous glucose monitoring systems and insulin pumps

* 1 non-empirical studies, e.g. discussion papers, recommendations, information and communications

2 empirical studies, e.g. surveys, analyses of insurance data or inpatient data
